# CHEMDNER: The drugs and chemical names extraction challenge

**DOI:** 10.1186/1758-2946-7-S1-S1

**Published:** 2015-01-19

**Authors:** Martin Krallinger, Florian Leitner, Obdulia Rabal, Miguel Vazquez, Julen Oyarzabal, Alfonso Valencia

**Affiliations:** 1 Structural Computational Biology Group, Structural Biology and BioComputing Programme, Spanish National Cancer Research Centre, Calle Melchor Fernndez Almagro, 3, Madrid, Spain; 2 Computational Intelligence Group, Department of Artificial Intelligence, Universidad Politecnica de Madrid, Calle Ramiro de Maeztu, 7, Madrid, Spain; 3 Small Molecule Discovery Platform, Center for Applied Medical Research (CIMA), University of Navarra, Avenida de Pio XII, 55, Pamplona, Spain

**Keywords:** named entity recognition, BioCreative, text mining, chemical entity recognition, machine learning, chemical indexing, ChemNLP

## Abstract

Natural language processing (NLP) and text mining technologies for the chemical domain (*ChemNLP *or chemical text mining) are key to improve the access and integration of information from unstructured data such as patents or the scientific literature. Therefore, the BioCreative organizers posed the CHEMDNER (chemical compound and drug name recognition) community challenge, which promoted the development of novel, competitive and accessible chemical text mining systems. This task allowed a comparative assessment of the performance of various methodologies using a carefully prepared collection of manually labeled text prepared by specially trained chemists as Gold Standard data. We evaluated two important aspects: one covered the indexing of documents with chemicals (**chemical document indexing **- *CDI *task), and the other was concerned with finding the exact mentions of chemicals in text (**chemical entity mention recognition **- *CEM *task). 27 teams (23 academic and 4 commercial, a total of 87 researchers) returned results for the CHEMDNER tasks: 26 teams for CEM and 23 for the CDI task. Top scoring teams obtained an F-score of 87.39% for the CEM task and 88.20% for the CDI task, a very promising result when compared to the agreement between human annotators (91%). The strategies used to detect chemicals included machine learning methods (e.g. conditional random fields) using a variety of features, chemistry and drug lexica, and domain-specific rules. We expect that the tools and resources resulting from this effort will have an impact in future developments of chemical text mining applications and will form the basis to find related chemical information for the detected entities, such as toxicological or pharmacogenomic properties.

## Background

Unstructured data repositories contain fundamental descriptions of chemical entities, such as their targets and binding partners, metabolism, enzymatic reactions, potential adverse effects and therapeutic use, just to name a few. Being able to extract information on chemical entities from textual data repositories, and particularly the scientific literature, is becoming increasingly important for researchers across diverse chemical disciplines [1]. Manual curation of papers or patents to generate annotations and populate chemical knowledgebases is a very laborious process that can be greatly improved through the use of automated language processing pipelines. Text-mining methods have shown promising results in the biomedical domain, where a considerable amount of methods and applications have been published [2,3]. These attempts cover tools to rank articles for various topics of relevance [4], detect mentions of bio-entities [5,6], index documents with controlled vocabulary terms [7] or even extract complex relationships between entities like physical protein-protein interactions [8]. Automatically transforming recognized entity mentions into structured annotations for biomedical databases has been studied in particular for genes or proteins [9].

Linking chemical entities to the results obtained by biological/biomedical text mining systems requires first the automatic recognition and indexing of chemical entities in documents. Furthermore, knowing which compounds are described in a given paper, and where exactly those descriptions are, is key to select appropriate papers. Only with such fine-grained annotations, it is possible to directly point to relevant sentences and to extract more detailed chemical entity relations. The process of automatically detecting the mentions of a particular semantic type in text is known as named entity recognition (NER). Some of the first NER systems constructed where those that recognized entities from newswire texts. Among the classical entities detected by such domain-independent tools are names of persons, organizations or locations [10]. In the context of biomedical literature mining, the bio-entities that attracted more interest were genes, proteins, cell lines, cell types, drugs, mutations and organisms or species [11,1,14]. The recognition of gene and protein mentions was addressed in several community challenges (BioCreative I, II, JNLPBA) that served to determine the state of the art methodology and systems performance [5,12] in addition of providing valuable datasets for developing new systems [15].

The recognition of chemical entities has to cope with a considerable degree of naming variability encountered between and within different chemical sub-disciplines. A given chemical entity can appear in the literature as a trivial or trademark name of a drug, as a short form (abbreviation or acronym), or it can be referred to in text following standard naming nomenclature guidelines as provided by the IUPAC. In addition, alternative typographical expressions for chemical entity mentions and ambiguity of chemical names that can correspond to other entity types (depending on the context of mention) makes detection of chemical names a demanding task.

Manually annotated text collections that were exhaustively labeled with entity mentions are essential to implement NER tools, especially if they rely on statistical machine learning methods. The lack of publicly available, large, and manually annotated text corpora for chemical entities was one of the main reasons why only few chemical compound recognition applications were available [1] before the CHEMDNER task. Details on the construction of high quality text corpora for chemistry, together with the underlying selection criteria and guidelines are presented in the CHEMDNER corpus paper of this same special issue.

We have organized the CHEMDNER task as part of the BioCreative community challenge (BioCreative IV [16]), to promote the development of systems for the automatic recognition of chemical entities in text. The CHEMDNER task was the first community-wide attempt to evaluate chemical natural language processing methods. It offered manually annotated data that allowed participating researches to improve and evaluate their tools. This task could help to increase the performance of chemical NER systems and run a comparative assessment across heterogeneous strategies. A number of participating teams present updated versions of their systems in this special issue, showing that the CHEMDNER task was a successful environment to implement their systems and that they could actually further improve their tool by learning from other participating approaches. This article will present the obtained results and provides an overview of the methods used by participating teams.

## Methods

### Task description

We divided the BioCreative IV CHEMDNER track into two tasks, each of practical relevance for the retrieval and extraction of chemical information from the literature. One task was concerned with the ability to determine which compounds appear in a given document, a requirement for selecting articles that refer to a particular chemical of interest. Therefore, this assignment is called the *chemical document indexing *(CDI) task. For the CDI task, given a collection of PubMed abstracts, participants had to provide for each of them a non-redundant (unique) list of chemical entities. Chemical entities were defined for the CDI task as the (UTF-8 encoded) character strings found in the text. The entities had to be returned as a ranked list to express the entity's likelihood of being a relevant chemical mention in that document, together with a confidence score. Each entity mention in the list had to be unique (for a given document). Submissions containing multiple ranks for the same chemical name were discarded. Although of great practical importance, we did not require that the returned compounds had to be mapped to their chemical structures or database identifiers. Normalization - or grounding - of entities to a knowledgebase is particularly challenging in case of chemicals [17], because many compounds found in literature (and patents) do not have any corresponding record in open access chemical databases. This means that only a subset of the compound mentions can be linked to an existing database. However, we consider the conversion of chemical names to structures a task of its own, more closely related to the cheminformatics domain. Therefore, we decided to not confound this research problem with the task of detecting chemicals in text.

The other CHEMDNER task was concerned with the ability to specifically locate exactly within a document every chemical entity mention, defined as their start and end character indices (counting the position of the characters in the document spanning the mention of a chemical). We called this the *chemical entity mention recognition *(CEM) task, a key step for any further chemical relation mining approaches or to induce chemical lexicons from the literature automatically. To specify mentions of entities, one popular option is to require labeling of individual tokens (mostly word units), as was done for the first gene mention task of the BioCreative challenge [18]. In case of the biomedical literature, several different tokenization strategies have been tested [19] and also specialized tokenizers have been proposed [20]. Tokenization of chemical literature is even more difficult, mainly due to the variable use of hyphens, parenthesis, brackets, dashes, dots and commas. We therefore did not pre-impose any tokenization of the CHEMDNER text collection and defined entities only at the character offset level, similarly to the last gene mention recognition task of BioCreative [5].

For both tasks, teams had to provide ordered results (ranked entity names or ranked entity mention offsets) together with confidence scores that reflected how sure they were that the extracted entity was correct. This setting promoted the implementation of systems that are more efficient for the manual validation of automatically extracted results. It facilitates selecting any N top results for each document. All the chemical annotations and predictions for both tasks were derived exclusively from the PubMed titles and abstracts; information from full text articles was not annotated for the CHEMDNER tasks. Participating teams could submit up to five runs for each of the two tasks. It was not mandatory to send predictions for both tasks; they could send results for any of the two or for both the CDI and CEM tasks. One strict constraint posed to participating teams was that any manual (human) correction or adjustment of the official results that they submitted for the test set documents were forbidden (i.e. only fully automated results were allowed). Compliance with the CHEMDNER prediction format was checked by the BioCreative evaluation script that was distributed to assess consistency and performance of automated predictions [8]. Table 1 shows two example team predictions for each of the two tasks. In that table, the first column is the PubMed identifier (PMID) and each line corresponds to one prediction for that document. In case of the CDI task, the second column contains the unique chemical entity mention string. For the CEM task, it corresponds to the chemical mention offset, specified as the part of the document record (T: Title, A: abstracts) followed by the offset of the starting character and the ending character of the mention span (separated by ':'). The third column (for the CDI and CEM predictions) corresponds to the rank for each prediction given the article. The fourth column of each task prediction contains the confidence score (Conf.). In these examples, only the top ten predictions per task and for the article with the PubMed identifier 23380242 are shown.

**Table 1:** Example team predictions for the CDI (left) and CEM (right) tasks.

	CDI			CEM		
PMID*	**Chemical**	**Rank**	**Conf**.	**Offset**	**Rank**	**Conf**.

23380242	TiO2	1	0.9	T:0:16	1	0.5
23380242	Titanium dioxide	2	0.9	A:323:331	2	0.5
23380242	pyrrolidine dithiocarbamate	3	0.9	A:333:337	3	0.5
23380242	SB203580	4	0.9	A:528:532	4	0.5
23380242	titanium	5	0.9	A:763:767	5	0.5
23380242	LY294002	6	0.9	A:894:898	6	0.5
23380242	epigallocatechin gallate	7	0.9	A:945:949	7	0.5
23380242	apocynin	8	0.9	A:1108:1112	8	0.5
23380242	PD98059	9	0.9	A:1118:1122	9	0.5
23380242	SP600125	10	0.9	A:1342:1369	10	0.5

Both task predictions have as first column the document identifier (PMID) and originally are formatted as tabulator-separated plain text columns. Only the top ten predictions are shown.

### Task data: CHEMDNER corpus

The predictions generated by automated systems were compared against manually labeled annotations done by domain experts. This manually labeled collection of texts and annotations is called the CHEMDNER corpus. The task was temporally structured into four periods, associated with the release of the CHEMDNER corpus datasets (refer to [21] for a detailed description of the corpus). The pre-release phase was a period before the actual data release, during which we distributed the initial annotation guidelines together with an annotated sample set. During the training phase, teams could explore the annotated training data to build their systems. Thereafter the development set was released, consisting of additional annotated abstracts useful for the evaluation and improvement of the participating systems. Finally, during the test set prediction phase, registered teams were provided with a collection of articles without annotations for which they had to return predictions within a short period of time, together with a technical system description. The entire CHEMDNER corpus consisted of a collection of 10,000 recently published PubMed abstracts representative of various chemistry-related disciplines. All abstracts were exhaustively annotated for chemical entity mentions by trained chemistry domain experts with experience in literature curation. The annotation process followed carefully defined annotation guidelines of rules for defining what actually was considered as a chemical entity and what not, as well as how to determine the individual mention boundaries of a chemical entity in text. As a minimum criteria, chemical entities had to correspond to chemical names that could be potentially linked to a chemical structure, excluding very general chemical terms and very large macromolecular entities, such as proteins. Additionally the annotations were manually classified into one of the following chemical mention classes: abbreviation (short form of chemical names including abbreviations and acronyms), formula (molecular formulas), identifier (chemical database identifiers), systematic (IUPAC names of chemicals), trivial (common names of chemicals and trademark names), family (chemical families with a defined structure) and multiple (non-continuous mentions of chemicals in text). These more granular annotation types should help participants to adapt their entity recognition strategies for particularities specific to each of the chemical entity mention classes. The entire CHEMDNER corpus was randomly sampled into three subsets, the training set (3,500 abstracts), development set (3,500 abstracts) and test set (3,000 abstracts). Overall, the CHEMDNER corpus contained 84,355 chemical entity mentions corresponding to 19,806 unique chemical names. The fraction corresponding to the test set used for evaluation purposes had 25,351 chemical entity mentions (7,563 unique chemical names).

### Evaluation metrics

Participating systems were permitted to integrate previously accessible third party tools and lexical resources relevant to chemistry in addition to the official CHEMDNER annotations. To keep the task simple, we did not request predictions of the class of chemical entity mentions (e.g. systematic, trivial etc.). We provided an evaluation script together with the data that calculated the performance of predictions against the Gold Standard data and returned all the evaluation scores that were used for the CHEMDNER task.

The metrics used for scoring the predictions were micro-averaged recall, precision and F-score. The balanced F-score was the main evaluation metric used. Three result types were examined: False negative (FN) results corresponding to incorrect negative predictions (cases that were part of the Gold Standard, but missed by the automated systems), False positive (FP) results being cases of incorrect positive predictions (wrong results predicted by the systems that had no corresponding annotation in the Gold Standard) and True positive (TP) results consisting of correct positive predictions (correct predictions matching exactly with the Gold Standard annotations). We did not examine partial hits of predictions that only in part overlapped with the manually defined Gold Standard annotations.

Recall *r *is the percentage of correctly labeled positive results over all positive cases.

(1) $$r:=\frac{TP}{TP+FN}$$

It is a measure of a systems ability to identify positive cases.

Precision *p *is the percentage of correctly labeled positive results over all positive labeled results.

(2) $$p:=\frac{TP}{TP+FP}$$

It is a measure of a classifier's reproducibility of the positive results.

The F-measure *F_β _*is the harmonic mean between precision and recall, where *β *is a parameter for the relative importance of precision over recall.

(3) $$F_{\beta }:=\left(1+\beta^{2}\right)\frac{p\cdot r}{\beta^{2}p+r}$$

The balanced F-measure (*β *= 1, referred to as "F-score" in this work) can be simplified to:

(4) $$F_{1}:=2\frac{p\cdot r}{p+r}$$

## Results

We received predictions from a total of 27 teams for the CHEMDNER challenge: 26 for the CEM task and 23 for the CDI task. In total, 87 researchers took part in participating teams, most of the teams were affiliated to academic research institutions but also 4 commercial teams submitted predictions. Table 2 provides an overview of participating teams, the tasks for which they submitted results (number of runs and the rank of their best submission grouped into subsets based statistical significance between them) and the link to the corresponding software in case it is available. A number of teams published a systems description paper in this same special issue of the Journal of Chemoinformatics. Those papers provide additional details on the used methods as well as potential updates and improvements of the initial approach that was used for the task. Total of 91 submissions for the CDI and 106 submissions for the CEM were evaluated. For properly interpreting text mining results, it is important to put automated systems performance into context. A simple baseline for detecting entity mentions is to label those mentions in the test set that have been seen before in the training data. A widespread baseline approach for NER methods is the *vocabulary transfer*, defined as the proportion of entities (without repetition) that are present both in the training/development data as well as in the test corpus. The vocabulary transfer is an estimate of the lower boundary expected recall of automatic NER methods. In previous assessments, for instance in MUC-6 (Palmer & Day) it was 21%, while in case of the CHEMDNER task it was of 36.34% when using both the training and development set names and of 27.77% when using only the names from the training set. We generated a dictionary-lookup baseline system that used the chemical entity list derived from the training and development set to tag the test set abstracts. For the CDI task, this strategy obtained a micro-averaged F-score of 53.85% (with a precision of 53.71% and recall of 54.00%), while in case of the CEM task it reached a micro averaged F-score of 57.11 (precision of 57.22%, recall of 57.00%). These scores indicate that there are some frequently mentioned chemical compounds in PubMed that can be exploited for labelling text, but also that many of them are ambiguous and just using dictionary look-up is not enough to capture the novel compound mentions. The upper boundary of named entity recognition performance is commonly measured by comparing independent annotations carried manually by human curators. The resulting value, called inter-annotator agreement (IAA) or inter-coder agreement score is useful to assess how well the task is defined, how consistent the annotations are and it helps to quantity the difficulty of the annotation process. The simplest IAA score is based on the percentage agreement of manual annotations between two different annotators. In case of the CHEMDNER corpus the percentage agreement between curators for defining chemical entity mentions was of 91% (exact matches) based on manual annotations of 100 abstracts. Additional details on the chemical annotation consistency analysis and IAA are provided in the CHEMDNER corpus paper [21].

**Table 2:** CHEMDNER team overview.

Id	Team contact	Type	Affiliation	CDI	CEM	**Ref**.	Tool URL
173	Z. Lu	A	NCBI/NLM/NIH, USA	5/3	5/1	[22]*	[23]

177	T. Can	A	Middle East Technical Univ., Ankara, Turkey	2/16	2/16	[24]	[25]

179	D. Lowe	C	NextMove Software	5/4	5/2	[26]*	[27]

182	A. Klenner	A	Fraunhofer-Institute for Algor. and Sci.Comp., Germany	4/17	4/18	-	-

184	R. Rak	A	National Centre for Text Mining; Univ. Manchester, UK	5/1	5/3	[28]*	-

185	S.V. Ramanan	C	RelAgent Pvt Ltd	3/6	3/6	[29]	[30]

191	A. Usie Chimenos	A	Univ. of Lleida, Spain	3/15	1/17	[31]*	[32]

192	H. Xu	A	Univ. of Texas Health Science Center at Houston, USA	0/-	5/5	[33]*	-

196	F. Couto	A	LASIGE, Univ. of Lisbon, Portugal	5/15	5/14	[34]*	[35]

197	S. Matos	A	Univ. of Aveiro, Portugal	5/5	5/4	[36]*	[37]

198	P. Thomas	A	Humboldt-Univ. zu Berlin, Germany	5/2	5/3	[38]	[39]

199	M. Irmer	C	OntoChem	1/8	1/7	[40]	[41]

207	K. Verspoor	A	National ICT Australia, Australia	2/10	2/11	[42]	-

214	D. Bonniot de Ruisselet	C	ChemAxon	5/9	5/8	-	[43]

217	L. Li	A	Dalian Univ. of Technology, P.R. China	5/11	5/14	[44]	-

219	M. Khabsa	A	The Pennsylvania State University, USA	5/13	5/12	[45]*	[46]

222	S.A. Akhondi	A	Erasmus MC, Rotterdam, The Netherlands	5/8	5/7	[47]*	-

225	D. Sanchez-Cisneros	A	Univ. Carlos III and Univ. Autonoma Madrid, Spain	5/18	5/19	[48]	[49]

231	D. Ji	A	Wuhan University, China	5/1	5/1	[50]*	[51]

233	T. Munkhdalai	A	Chungbuk National Univ., South Korea	5/4	5/5	[52]*	[53]

238	H. Liu	A	Mayo Clinic, USA	5/14	5/13	[54]	-

245	S. Zitnik	A	Univ. of Ljubljana, Slovenia	3/7	3/7	[55]	-

259	S. Xu	A	Inst. of Scien. and Techn. Info. of China, P.R. China	0/-	5/7	[56]*	[57]

262	A. Ekbal	A	IIT Patna, India	0/-	5/9	[58]	-

263	M. Yoshioka	A	Hokkaido Univ., Sapporo, Japan	0/-	3/10	[59]	-

265	S. Ching-Yao	A	Yuan Ze Univ./Taipei Medical Univ., Taiwan, R.O.C.	2/13	2/15	[60]	-

267	L. Li	A	Dalian Univ. of Technology, P.R. China	1/12	0/-	-	-

In the CDI and CEM columns the number or runs and the rank group of the best submission are shown. The asterisks indicate teams with articles in this special issue. Id: team identifier, A: academic, C: commercial

To examine the statistical significance of each prediction with respect to other submissions, we carried out a Bootstrap resampling simulation in a similar way to what was previously done for the gene mention task of Biocreative II [5]. This statistical analysis was done for both the CDI and CEM tasks by taking 1,000 bootstrapped samples from all 2,478 articles in the test set that had annotations. We then calculated the micro-averaged F-scores for each team on each sample. These 1,000 resampled results were then used to calculate the standard deviation of each teams F-score (SDs). The evaluation tables of the CDI and CEM tasks illustrate the range of other teams (rows) that are covered within two standard deviations of a teams own F-score (Range). We used this range to group teams that have no statistically significant difference (at two SD) between results (Group).

We received 91 runs from the 23 teams that participated in the CDI task. The evaluation of the best performing test set predictions from each team against the manual Gold Standard annotations are shown in table 3 (for the complete list of results for all CDI runs refer to additional materials table 1 i.e. Additional file 1). The table 3 shows the micro-averaged precision (P), recall (R), and balanced F-score (F1) for the highest-scoring runs/team. The top-ranking F-score obtained for the CDI task was of 88.20% by team 231 (run 3) very closely followed by run 3 from team 184 (F-score of 88.15%). There was no significant difference between these two top scoring runs. Eight teams had predictions with CDI prediction F-scores above 80%. When looking at the precision and recall performance separately, the highest precision obtained by a participant was 98.66% (with a modest recall of 16,65%) while the highest recall was of 92.24% (with a precision of 76.29%). In general the precision scores of the team submissions were slightly better than the corresponding recall.

**Table 3:** CDI evaluation results (best run per team only).

Row	Team	*P*	*R *	*F*_1_	*SD_s_*	Range	Group	Rank
A	231	87.02%	89.41%	88.20%	0.30%	A-C	1	1
B	184	91.28%	85.24%	88.15%	0.34%	A-C	1	2
C	198	89.34%	86.51%	87.90%	0.33%	A-D	2	3
D	173	87.81%	87.24%	87.52%	0.33%	C-D	3	4
E	179	87.58%	84.86%	86.20%	0.36%	E-F	4	5
F	233	86.03%	85.45%	85.74%	0.35%	E-F	4	6
G	197	86.35%	82.37%	84.31%	0.35%	G-G	5	7
H	185	82.77%	83.19%	82.98%	0.38%	H-H	6	8
I	245	83.35%	75.38%	79.17%	0.41%	I-I	7	9
J	199	84.91%	71.46%	77.61%	0.46%	J-K	8	10
K	222	84.55%	71.65%	77.57%	0.46%	J-K	8	11
L	214	86.40%	68.77%	76.58%	0.47%	L-M	9	12
M	207	81.24%	71.07%	75.82%	0.46%	L-N	10	13
N	217	73.44%	77.25%	75.30%	0.42%	M-N	11	14
O	267	72.65%	75.86%	74.22%	0.45%	O-O	12	15
P	219	79.11%	66.13%	72.04%	0.46%	P-Q	13	16
Q	265	83.85%	62.40%	71.55%	0.48%	P-Q	13	17
R	238	76.49%	64.96%	70.25%	0.56%	R-R	14	18
S	191	80.99%	58.28%	67.78%	0.49%	S-T	15	19
T	196	57.66%	81.48%	67.53%	0.38%	S-T	15	20
U	177	62.11%	70.20%	65.91%	0.47%	U-U	16	21
V	182	60.31%	57.36%	58.79%	0.49%	V-V	17	22
W	225	60.80%	53.09%	56.69%	0.50%	W-W	18	23

*P*: precision; *R*: recall, *F*_1_: F-score, *SD_s_*: standard deviation in the bootstrap samples.

The best result of the CEM task was marginally below the top result of the CDI task. The best scoring prediction (by team 173, run 2) obtained an F-score of 87.39, closely followed by team 231 with 87.11% (see table 4). These scores can be considered already competitive results taking into account the underlying IAA of 91% of the annotations. It is also important to keep in mind that this was the first time that such a task was carried out and that teams had a rather short timeframe to build/train their tools, indicating that these initial results could be further improved. Nine teams obtained an F-score above 80% and the highest precision of a submission was of 98.05 (recall 17.90). The top recall of systems for the CEM task was of 92.11 (with a precision 76.72). The complete list of evaluated CEM results can be seen in the additional materials table 2 (Additional file 2). Overall precision scores, when compared to their corresponding recall values were considerable better in case of the CEM task (even more than in case of the CDI task). This might indicate that there is still some room for improving the overall recall of participating systems. To better understand issues related to the chemical entity recognition recall we examined the subset of entities that were only present in the test set and did not have any previous mention neither in the training nor development set (novel chemical mentions). The highest recall of such novel chemical mentions was of 83.49% (team 173, run 3), more than eight percent lower than the recall on the entire test set mentions.

**Table 4:** CEM evaluation results (best run per team only).

Row	Team	*P*	*R*	*F*_1_	*SD_s_*	Range	Group	Rank
A	173	89.09%	85.75%	87.39%	0.37%	A-C	1	1
B	231	89.10%	85.20%	87.11%	0.37%	A-C	1	2
C	179	88.73%	85.06%	86.86%	0.41%	A-E	2	3
D	184	92.67%	81.24%	86.58%	0.39%	C-F	3	4
E	198	91.08%	82.30%	86.47%	0.38%	C-F	3	5
F	197	86.50%	85.66%	86.08%	0.45%	D-F	4	6
G	192	89.42%	81.08%	85.05%	0.44%	G-H	5	7
H	233	88.67%	81.17%	84.75%	0.40%	G-H	5	8
I	185	84.45%	80.12%	82.23%	0.44%	I-I	6	9
J	245	84.82%	72.14%	77.97%	0.47%	J-N	7	10
K	199	85.20%	71.77%	77.91%	0.47%	J-N	7	11
L	222	85.83%	71.22%	77.84%	0.50%	J-N	7	12
M	259	88.79%	69.08%	77.70%	0.49%	J-N	7	13
N	214	89.26%	68.08%	77.24%	0.52%	J-O	8	14
O	262	78.28%	74.59%	76.39%	0.45%	N-P	9	15
P	263	82.14%	70.94%	76.13%	0.46%	O-P	10	16
Q	207	84.63%	67.48%	75.09%	0.51%	Q-Q	11	17
R	219	80.46%	67.54%	73.44%	0.51%	R-R	12	18
S	238	76.90%	66.65%	71.41%	0.56%	S-S	13	19
T	196	63.92%	77.88%	70.21%	0.44%	T-U	14	20
U	217	73.17%	67.23%	70.08%	0.47%	T-U	14	21
V	265	86.35%	57.17%	68.79%	0.49%	V-V	15	22
W	177	62.23%	67.84%	64.92%	0.55%	W-W	16	23
X	191	75.71%	55.04%	63.74%	0.59%	X-X	17	24
Y	182	61.46%	60.33%	60.89%	0.57%	Y-Y	18	25
Z	225	62.47%	53.51%	57.65%	0.59%	Z-Z	19	26

*P*: precision; *R*: recall, *F*_1_: F-score, *SD_s_*: standard deviation in the bootstrap samples.

Additionally, we also did a more granular evaluation of the recall for each of the individual chemical entity mention classes. The results for each class and the novel mentions are shown in additional materials table 3 (Additional file 3). The best results were obtained for the *systematic *class, corresponding to the names that follow the chemical nomenclature standards (IUPAC or IUPAC-like chemical names), with a top recall of 95.89%. Although correctly identifying mention boundaries of systematic names can be difficult, such kind of mentions do also show very strong word morphology and character n-grams characteristics distinct from other surrounding words. The second best recall result was obtained for trivial names, where one team reached a 94.25%. Trivial chemical names are better covered by lexical resources containing extensive lists of generic drug names and also drug brand names. They also do show some particularities that can be detected by machine learning methods like the usage of typical stems and affixes which denote characteristics of drugs (e.g. mode of action or the class a drug belongs to, e.g. -vir for antiviral drugs or -tinib for tyrosine kinase inhibitors). The best recall for other types of chemical entity classes was slightly worse, being 91.38% in case of chemical abbreviations and 90.06% for both identifiers and chemical families, followed by chemical formula with a recall of 89.37%. These types of chemical entities do often have a higher degree of ambiguity, especially some acronyms and short formula. The most problematic class was the chemical class *multiple*. Where the highest recall was of only 60.30%. In case of the CHEMDNER corpus those mentions account for less than one percent of the total (0.78%) number of chemical mentions. To determine the difficulty of each chemical mention class we examined how many runs correctly identified each of the Gold Standard chemical mentions and then looked at what chemical class it belonged to. Only 108 of the 25,351 test set chemical mentions were not detected by any of the teams, implying that over 99.99% of the mentions could be retrieved by at least one team. The Additional file 4 contains a figure that shows a boxplot with the number of runs that correctly identified each of the chemical entity mentions for each of the CEM classes. This figure indicates that trivial mentions on the whole, were the easiest ones for the participants followed by systematic chemical mentions. From the other CEM classes, abbreviations and formula are two types of mentions that do account for an important number of mentions in the test set and for which it is clear that overall a number of systems would require a better recognition strategy. One common characteristic of all participating teams was that they all used the provided CHEMDNER corpus either to train their system or to adapt and fine-tune previously implemented software. Only five teams also utilised other external corpora. These teams obtained on average a slightly worse F-score of 72.74% compared to the teams that only used the CHEMDNER corpus (F-score of 77.44%). Most of the participants (22 teams) used the official evaluation library to validate and improve their systems during the training and development phase. Those teams on average also obtained superior results (F-score of 77.36%) when compared to teams that did not rely on the BioCreative evaluation script (F-score of 71.99%).

## Discussion

The CHEMDNER task of BioCreative IV showed that the automatic recognition of chemical entities from PubMed abstracts is a feasible task by automated named entity recognition systems. The only mention class that still requires clearly a better detection performance is the class *multiple*, where individual entities do not correspond to a non-continuous string of text. A more fine-grained annotation of this particular class of mention together with the annotation of the actual dependencies of the various text strings that do correspond to a chemical could help to improve their detection. Another problematic case is short chemical formulas (e.g.: I, O, P, H), as some are highly ambiguous and do correspond in most of the cases to nonchemicals. Despite the good results for trivial and systematic mentions of chemicals, examining some of the frequent false negative cases not detected by many of the participating teams showed that there were also some common difficulties. Teams had problems in finding trivial names corresponding to dyes. Trivial names that showed unusual word morphology with embedded brackets were hard in terms of the correct mention boundary recognition. The only obvious issue with systematic names was encountered for very long names, those were challenging in terms of the correct mention boundary detection. Also some of the systems did apply a length cut-off when detecting chemicals, especially those that relied chemical dictionary lookup as the recognition approach. We carried out a survey on participating teams to better summarize the most relevant aspects of participating techniques. In this survey we covered aspects such as the used methodologies, exploited resources and software as well as the underlying features for the detection of chemical mentions. Most of the teams used some sort of lexical resources (lists of chemical names) derived from various databases or terminologies. In particular, ChEBI, PubChem and DrugBank were the most commonly used lexical resources. Some of the top scoring teams did additionally also some automated expansion of these original lists of chemicals. Additional file 5 provides a compendium of the main resources explored by the CHEMDNER participants.

**Figure 1:**
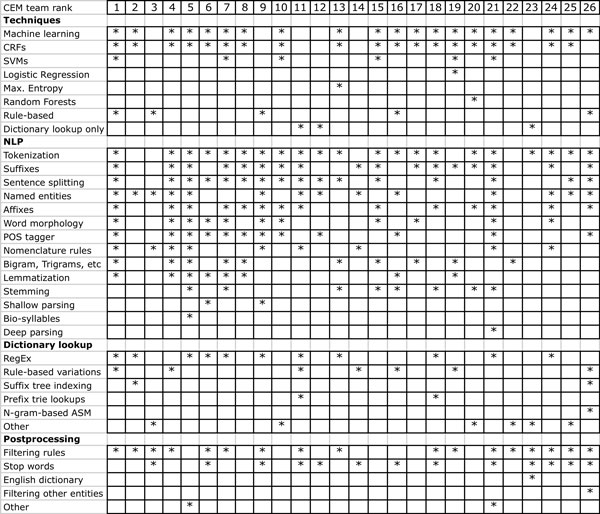
**Overview of the methods used by participating teams**.

The majority of teams did also explore existing chemical entity recognition software, mostly Oscar4 and ChemSpot. The output of external chemical entity taggers was frequently used as one more feature by participating systems. Some participants used only the text tokenization modules provided by these NER systems (for instance from Oscar4) as an alternative to more generic tokenization modules. Only few teams did use existing biomedical NLP/text mining software. For instance the top ranking team of the CEM task did integrate the AB3P (Abbreviation Plus P-Precision) tool for recognizing potential abbreviations of chemical names. As can be seen in Additional file 5, teams also adapted a range of packages that implement machine learning algorithms (e.g. Mallet or CRF++) or general natural language processing software (e.g. OpenNLP) to build their systems. The participants used three general strategies to identify chemical entity mentions: (1) supervised machine learning approaches, (2) rule/knowledge-based approaches and (3) chemical dictionary look-up approaches. Most of the systems were hybrid systems using machine learning techniques based on conditional random fields (CRFs) that exploited also chemical dictionaries as one of the used lexical features. Figure 1 provides a summary of the participating systems based on the responses provided in the CHEMDNER survey. Analyzing the used techniques, indicated that CRFs were the method of choice for most teams, and that this machine learning technique can be considered as an efficient strategy for chemical NER. Only few teams explored the used of other machine learning techniques, mainly SVMs, which were used additionally to CRFs by six teams. It's worth noting that two systems that used mainly rule-based methods (together with some chemical gazetteers lookup) could also obtain competitive results, ranking third (team 179) and ninth (team 185) in the CEM task.

**Figure 2:**
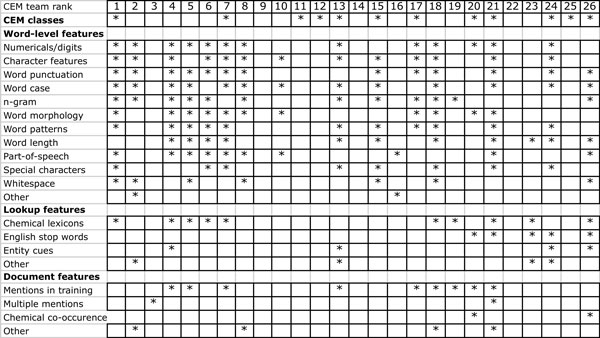
**Overview of used features by participating teams**.

Building of these rule-based systems required a deep understanding of both the existing chemical nomenclature standards as well as of the CHEMDNER annotation guidelines. Surprisingly, two systems relied essentially on the use of lexical resources for chemical names (team 199 and team 222), exploiting a considerable number of different databases and terminologies they could obtain satisfactory results (rank 11 and 12 in the CEM task). The use of dictionary-lookup based approaches required efficient dictionary pruning and post-processing as well as strategies to do expansion of the original lists of chemicals. The top scoring team of the CEM task was a hybrid strategy that integrated all three general methods, that is a machine learning based approach based on various CRF models, patterns to find special types of mentions such as chemical formula and sequences of amino acids as well as chemical gazetteers. It also integrated an abbreviation detection method. Although this system did not explore more sophisticated chemical nomenclature rules and also made use of a limited chemical dictionary, the integration of those two additional methods served to improve the overall performance of this system when compared to other participants. We would expect that by combining CRFs-based models with sophisticated rule based approaches such as the one used by team 179 and extensive lexical resources such as explored by teams 199 and 222 would further improve chemical entity detection results. Analyzing the top-performing machine learning based systems also indicated that they did carefully examine various text tokenization modules and that the use of chemical domain-specific tokenization could slightly improve their results. They also used nomenclature rules as features and carried out extensive automatic post-processing of the results (e.g. checking if brackets are balanced within the chemical name). A more detailed examination of the various featured used by participating systems can be seen in Figure 2.

Teams could submit up to five runs for each of the tasks. Examining at a general level, the differences between the returned runs showed that they corresponded to either different (or different combinations) of the used CRF models or were runs optimized for recall, precision or F-score.

We requested the submission of a confidence score and ranking of the predicted mentions/chemical entities, but we did not carry out a proper evaluation of the ranking this time, mainly because the used documents (abstracts) were too short to do a meaningful analysis. The rankings fro the CDI task were generated by participants using various alternatives: (1) simply counting the number of occurrences of mentions, (2) using manual rules to define the ranking based on the chemical entity class, (3) ranking the mention by examining if they were present in some chemical lexicon (e.g. ChEBI), (4) checking if the extracted mention was present in the training/development set, (5) use of confidence scores or marginal probabilities provided by the machine learning models.

Almost all systems relied on essentially the same pipeline for both the CEM and CDI task, returning the results of the CEM task (after filtering duplicate names and doing an entity ranking) as prediction for the CDI task. Therefore we thus think that doing a CDI task again would only be meaningful if the chemical entities have to be normalized to chemical structures or databases and if larger documents (full text articles of patents) are used.

## Conclusion

The CHEMDNER task was the first attempt to systematically examine independently the performance of chemical entity recognition methods. It could attract a considerable number of participants from academia and industry and resulted in a range of new applications for the recognition of chemical entity mentions. The best performing teams could reach a performance close to what could be expected by chemical database curators when manually labeling the text. Although 18 teams worked previously on this or a related topic, the CHEMDNER task could attract new research groups interested in the recognition of chemicals in text. Most teams (19) considered that, given a training data such as the CHEMDNER corpus provided for this task, the recognition of chemical entities is a task with a medium degree of difficulty and they would be interested in participating in a similar task again in the future. The CHEMDNER task was able to determine the state-of the art of chemical entity recognition systems and also promoted the improvement in terms of performance when compared to previously published methods. We expect that the tools resulting from this challenge constitute a valuable building block for text mining and information extraction technologies linking chemicals to other entities of interest such as genes and proteins, or to extract other relevant associations of chemical compounds (e.g. physical binding or drug target interactions, chemical entity metabolism, therapeutic, adverse effect, reactions and reactants, etc). We foresee that the CHEMDNER task will promote research in chemical entity recognition in general, but also provide useful insights for better processing of other document collections such as noisy text (in particular patents) and full text articles.

## Competing interests

The authors declare that they have no competing interests.

## Authors' contributions

MK was responsible for the task definition and coordinated the corpus annotation and result evaluation. FL and MV helped define the task, the annotations, and the evaluation of the results. OR and JO were responsible for refining the annotation guidelines and supervised the annotation quality and results. AV supervised the entire task setting. All authors revised the manuscript.

## Supplementary Material

Additional file 1 Click here for file

Additional file 2 Click here for file

Additional file 3 Click here for file

Additional file 4 Click here for file

Additional file 5 Click here for file

## Declarations

We would like to acknowledge funding for this publication by the Innovative Medicines Initiative Joint Undertaking (IMI-eTOX, under Grant Agreement nr. 115002)

This article has been published as part of *Journal of Cheminformatics *Volume 7 Supplement 1, 2015: Text mining for chemistry and the CHEMDNER track. The full contents of the supplement are available online at http://www.jcheminf.com/supplements/7/S1.
