# Supplementary material for: CHEMDNER: The drugs and chemical names extraction challenge
Source: J Cheminform. 2015 Jan 19;7(Suppl 1):S1. doi: 10.1186/1758-2946-7-S1-S1 (PMC4331685; doi:10.1186/1758-2946-7-S1-S1)
Supplement: Additional file 1 [file 1758-2946-7-S1-S1-S1.pdf]

Additional file 1: CDI evaluation results for all teams and runs

Team	Run	Predictions	P	R	F_1
173	1	13382	88.036	86.056	87.035
173	2	13601	87.810	87.239	87.523
173	3	15215	82.675	91.885	87.037
173	4	13946	86.627	88.247	87.429
173	5	16552	76.293	92.243	83.513
177	1	16223	59.446	70.446	64.480
177	2	15473	62.108	70.197	65.905
179	1	12619	87.963	81.081	84.382
179	2	13265	87.584	84.865	86.203
179	3	12111	89.877	79.511	84.377
179	4	12790	89.163	83.302	86.133
179	5	13170	87.585	84.259	85.890
182	1	13020	60.307	57.356	58.794
182	2	64933	16.092	76.326	26.580
182	3	5092	92.321	34.339	50.059
182	4	2310	98.658	16.647	28.487
184	1	12977	90.383	85.676	87.966
184	2	12941	90.395	85.449	87.853
184	3	12784	91.278	85.237	88.154
184	4	12747	91.292	85.004	88.036
184	5	13360	88.518	86.384	87.438
185	1	13803	82.417	83.097	82.756
185	2	13807	82.400	83.104	82.751
185	3	13759	82.775	83.192	82.983
191	1	10375	74.361	56.355	64.118
191	2	9850	80.995	58.276	67.782
191	3	10428	76.592	58.342	66.233
196	1	0	0.000	0.000	0.000
196	2	1139	96.839	8.057	14.876
196	3	19346	57.655	81.476	67.526
196	4	3636	91.887	24.405	38.566
196	5	4838	77.139	27.261	40.285
197	1	13060	86.348	82.374	84.314
197	2	13456	83.851	82.418	83.128
197	3	12680	87.287	80.847	83.944
197	4	13120	84.619	81.096	82.820
197	5	12709	87.473	81.205	84.223
198	1	7961	85.127	49.503	62.602
198	2	13275	88.444	85.763	87.083
198	3	12988	88.251	83.725	85.928
198	4	13256	89.341	86.508	87.902
198	5	13188	89.134	85.866	87.469
199	1	11522	84.907	71.461	77.606
207	1	10344	77.369	58.459	66.597
207	2	11975	81.244	71.066	75.815
214	1	10267	86.199	64.646	73.882
214	2	10896	86.399	68.766	76.580
214	3	10422	88.582	67.436	76.576
214	4	12043	80.686	70.979	75.522
214	5	11568	82.694	69.876	75.746
217	1	13737	74.893	75.150	75.021
217	2	14400	73.444	77.253	75.301
217	3	14006	73.554	75.252	74.393
217	4	14667	72.155	77.305	74.641
217	5	13816	71.909	72.571	72.239
219	1	11444	79.107	66.129	72.038
219	2	40439	28.532	84.280	42.631
219	3	14589	66.975	71.373	69.104
219	4	5555	90.837	36.859	52.440
219	5	15880	63.054	73.141	67.724
222	1	12186	81.011	72.111	76.302
222	2	15578	66.074	75.186	70.336
222	3	12293	80.477	72.264	76.150
222	4	15830	65.193	75.383	69.919
222	5	11601	84.553	71.651	77.569

Continued on next page

Additional file 1 – continued from previous page

Team	Run	Predictions	P	R	F_1
225	1	11953	60.805	53.090	56.686
225	2	13349	56.948	55.530	56.230
225	3	23820	35.932	62.520	45.636
225	4	13973	53.603	54.711	54.152
225	5	22782	37.139	61.804	46.397
231	1	13051	89.411	85.237	87.274
231	2	14293	85.685	89.459	87.532
231	3	14066	87.018	89.408	88.197
231	4	12154	88.366	78.451	83.114
231	5	14582	84.453	89.956	87.118
233	1	13867	84.250	85.340	84.792
233	2	13495	83.134	81.950	82.538
233	3	13671	85.605	85.486	85.545
233	4	13522	83.153	82.133	82.640
233	5	13597	86.034	85.449	85.740
238	1	15737	56.898	65.405	60.856
238	2	25204	37.407	68.868	48.480
238	3	11627	76.486	64.960	70.253
238	4	17577	54.549	70.037	61.330
238	5	19968	47.907	69.876	56.842
245	1	12381	83.354	75.383	79.168
245	2	12783	81.147	75.771	78.367
245	3	13172	80.474	77.429	78.922
265	1	10122	83.393	61.658	70.897
265	2	10187	83.852	62.396	71.550
267	1	14294	72.653	75.858	74.221
