# Supplementary material for: CHEMDNER: The drugs and chemical names extraction challenge
Source: J Cheminform. 2015 Jan 19;7(Suppl 1):S1. doi: 10.1186/1758-2946-7-S1-S1 (PMC4331685; doi:10.1186/1758-2946-7-S1-S1)
Supplement: Additional file 2 [file 1758-2946-7-S1-S1-S2.pdf]

Additional file 2 : CEM evaluation results for all teams and runs

Team	Run	Predictions	P	R	F_1
173	1	25722	85.950	87.208	86.574
173	2	24402	89.087	85.752	87.388
173	3	28500	81.916	92.091	86.706
173	4	26514	85.155	89.062	87.064
173	5	30438	76.720	92.115	83.715
177	1	28804	59.881	68.037	63.699
177	2	27634	62.235	67.840	64.916
179	1	23098	89.216	81.287	85.067
179	2	24301	88.733	85.058	86.857
179	3	22270	90.813	79.776	84.937
179	4	23512	90.094	83.559	86.704
179	5	24118	88.689	84.375	86.478
182	1	24885	61.459	60.329	60.889
182	2	104925	19.708	81.571	31.746
182	3	9984	92.708	36.511	52.390
182	4	4627	98.055	17.897	30.269
184	1	22490	92.036	81.650	86.532
184	2	22378	92.023	81.232	86.291
184	3	22222	92.674	81.235	86.579
184	4	22109	92.664	80.813	86.334
184	5	23053	90.622	82.407	86.319
185	1	24097	84.218	80.052	82.082
185	2	24087	84.249	80.048	82.095
185	3	24051	84.454	80.123	82.231
191	1	18427	75.715	55.035	63.740
192	1	22924	89.138	80.604	84.657
192	2	22860	88.968	80.226	84.371
192	3	23021	89.279	81.074	84.979
192	4	22988	89.416	81.082	85.045
192	5	23503	88.304	81.867	84.963
196	1	0	0.000	0.000	0.000
196	2	1864	96.727	07.112	13.250
196	3	30886	63.922	77.879	70.214
196	4	6183	92.916	22.662	36.437
196	5	7952	79.779	25.025	38.099

Continued on next page

Team	Run	Predictions	P	R	F_1
197	1	25104	86.504	85.661	86.081
197	2	25694	84.747	85.894	85.317
197	3	24446	87.172	84.060	85.587
197	4	25102	85.352	84.513	84.931
197	5	24046	87.507	83.003	85.195
198	1	11696	87.278	40.267	55.108
198	2	22898	90.571	81.807	85.967
198	3	22201	90.136	78.936	84.165
198	4	22908	91.082	82.304	86.471
198	5	22630	90.968	81.204	85.809
199	1	21356	85.199	71.772	77.911
207	1	18121	80.145	57.288	66.815
207	2	20216	84.626	67.485	75.089
214	1	18819	87.082	64.644	74.204
214	2	20068	87.338	69.137	77.179
214	3	19334	89.262	68.076	77.243
214	4	22424	81.181	71.808	76.207
214	5	21711	82.903	70.999	76.491
217	1	22073	74.390	64.771	69.248
217	2	23291	73.174	67.228	70.075
217	3	22536	73.008	64.901	68.716
217	4	23751	71.858	67.323	69.517
217	5	22393	69.482	61.374	65.177
219	1	21281	80.461	67.544	73.439
219	2	71497	31.639	89.231	46.714
219	3	25536	72.024	72.549	72.286
219	4	9411	92.573	34.366	50.124
219	5	27506	68.720	74.561	71.521
222	1	22021	82.480	71.646	76.682
222	2	28592	66.938	75.496	70.960
222	3	22208	81.952	71.792	76.537
222	4	29010	66.115	75.658	70.565
222	5	21034	85.832	71.216	77.844
225	1	19662	65.924	51.130	57.592
225	2	21715	62.473	53.513	57.647
225	3	35974	41.922	59.489	49.184
225	4	22141	59.853	52.274	55.807
225	5	34563	42.826	58.388	49.411
231	1	22426	91.046	80.541	85.472
231	2	24497	88.199	85.227	86.688
231	3	24240	89.105	85.200	87.109
231	4	20900	90.450	74.569	81.745
231	5	24993	87.040	85.811	86.421
233	1	23486	87.371	80.944	84.035
233	2	22623	86.178	76.904	81.277
233	3	23284	88.464	81.251	84.704
233	4	22751	86.102	77.271	81.448
233	5	23206	88.671	81.168	84.754
238	1	28179	59.981	66.672	63.150
238	2	31725	56.328	70.490	62.618
238	3	35338	50.690	70.660	59.032
238	4	21972	76.902	66.652	71.411
238	5	45455	38.955	69.847	50.016
245	1	20438	86.657	69.863	77.359
245	2	21109	85.021	70.794	77.258
245	3	21562	84.821	72.143	77.970
259	1	17655	89.612	62.408	73.576
259	2	18538	89.174	65.208	75.331
259	3	19000	89.426	67.023	76.621
259	4	19457	89.058	68.352	77.343
259	5	19723	88.790	69.078	77.703
262	1	22900	77.629	70.123	73.686
262	2	22976	79.004	71.603	75.122
262	3	23565	77.322	71.875	74.499
262	4	24157	78.275	74.589	76.388
262	5	24347	76.149	73.133	74.611
263	1	20870	83.699	68.905	75.585
263	2	21425	82.856	70.025	75.902
263	3	21894	82.137	70.936	76.127

Continued on next page

Additional file 2 – continued from previous page

Team	Run	Predictions	P	R	F_1
265	1	16653	86.537	56.846	68.617
265	2	16786	86.346	57.173	68.795
