# Supplementary material for: CHEMDNER: The drugs and chemical names extraction challenge
Source: J Cheminform. 2015 Jan 19;7(Suppl 1):S1. doi: 10.1186/1758-2946-7-S1-S1 (PMC4331685; doi:10.1186/1758-2946-7-S1-S1)
Supplement: Additional file 3 [file 1758-2946-7-S1-S1-S3.pdf]

Additional file 3: CEM recall per team, run, and, class

Team	Run	Abbrev.	Family	Formula	Ident.	Multip.	System.	Trivial	Undef.	Novel
173	1	87.68	82.16	81.99	83.23	39.70	91.37	90.14	70.73	74.69
173	2	82.85	78.71	83.94	86.16	48.24	91.97	87.83	68.29	73.90
173	3	91.33	87.99	*89.37*	*90.06*	53.27	*95.89*	94.03	*78.05*	*83.49*
173	4	88.37	84.40	83.18	89.86	40.20	92.76	92.74	78.05	78.51
173	5	*91.38*	*90.06*	88.09	87.52	*60.30*	95.25	94.15	75.61	82.43
177	1	48.39	63.72	48.65	57.89	3.52	76.97	84.90	17.07	54.32
177	2	47.65	63.97	47.28	57.89	3.52	77.16	84.99	17.07	53.92
179	1	75.76	75.51	63.81	66.86	27.64	88.97	91.42	56.10	70.93
179	2	82.06	84.15	70.52	70.37	27.64	90.24	92.24	60.98	71.93
179	3	73.27	72.42	63.29	66.47	27.64	87.88	90.29	56.10	68.29
179	4	79.70	81.06	70.03	69.98	27.64	89.14	91.07	60.98	69.34
179	5	80.39	81.20	72.00	69.00	28.14	89.87	91.96	60.98	71.69
182	1	39.00	63.31	20.07	28.65	0.50	68.11	86.03	7.32	43.28
182	2	79.58	86.28	50.54	67.45	4.52	85.51	*94.25*	60.98	73.04
182	3	9.34	29.57	3.51	8.19	0.00	44.12	65.87	0.00	19.31
182	4	0.71	8.01	0.14	0.00	0.00	20.14	39.34	0.00	6.80
184	1	64.89	82.19	71.48	72.71	41.71	90.63	89.83	53.66	66.26
184	2	62.60	82.19	71.48	72.71	41.71	90.40	89.83	53.66	65.05
184	3	64.72	81.22	71.51	72.71	41.71	90.26	89.28	53.66	65.64
184	4	62.40	81.22	71.51	72.71	41.71	90.03	89.28	53.66	64.43
184	5	66.05	84.15	71.42	72.71	43.22	90.94	90.53	53.66	67.72
185	1	70.39	82.14	66.39	68.03	31.16	86.27	87.72	68.29	71.65
185	2	70.29	82.14	66.51	68.03	30.65	86.27	87.72	68.29	71.60
185	3	70.39	82.25	66.39	68.03	31.16	86.32	87.86	68.29	71.66
191	1	49.72	48.87	43.22	49.90	1.00	58.42	65.29	24.39	22.43
192	1	65.16	79.38	75.08	60.82	40.70	87.79	88.90	51.22	56.79
192	2	64.99	79.24	75.02	60.62	38.19	87.12	88.41	51.22	56.05
192	3	65.53	79.29	75.08	64.91	38.69	87.80	90.03	51.22	57.87
192	4	65.29	79.27	75.17	64.72	39.70	87.79	90.16	51.22	57.83
192	5	66.22	81.34	73.80	65.89	28.64	88.79	91.30	58.54	59.05
196	1	0.00	0.00	0.00	0.00	0.00	0.00	0.00	0.00	0.00
196	2	3.25	7.26	4.65	0.00	0.50	5.12	12.26	0.00	2.20
196	3	57.21	85.81	59.98	49.32	42.21	86.32	89.66	46.34	57.00
196	4	14.41	29.82	17.95	1.17	10.05	24.09	26.52	0.00	8.25
196	5	15.50	33.74	18.36	1.17	10.55	27.29	29.29	2.44	10.44
197	1	83.07	82.72	78.54	78.75	50.75	90.58	89.46	53.66	71.64
197	2	83.62	83.21	78.68	79.14	50.75	90.34	89.78	53.66	71.33
197	3	79.63	81.69	76.21	71.93	45.73	90.01	88.55	51.22	68.19
197	4	80.81	82.50	76.47	72.90	45.73	89.96	88.90	51.22	68.29
197	5	75.49	80.92	75.86	76.41	47.24	89.22	88.01	53.66	66.85
198	1	31.21	50.19	61.17	53.61	29.14	41.23	29.80	51.22	35.13
198	2	69.62	79.51	77.78	65.69	41.71	88.53	88.32	56.10	63.98
198	3	63.88	77.42	76.24	57.70	33.67	87.17	85.36	53.66	62.20
198	4	69.94	79.54	79.84	61.21	47.24	89.90	88.00	56.10	65.32
198	5	68.42	77.69	77.90	62.77	42.21	88.88	87.70	56.10	64.54
199	1	69.33	69.60	45.11	38.79	1.00	77.99	85.60	7.32	48.80
207	1	58.78	33.16	52.54	22.42	12.06	68.80	65.13	12.20	41.31
207	2	58.71	65.68	56.72	22.81	9.04	78.10	74.67	7.32	48.33
214	1	47.82	57.07	54.60	0.58	0.50	69.66	83.86	2.44	35.25
214	2	54.17	64.91	59.19	22.42	1.00	73.05	85.54	9.76	35.75
214	3	54.10	61.82	59.19	22.42	1.00	71.62	84.61	9.76	35.40
214	4	60.90	68.47	60.41	44.64	1.00	74.30	86.16	9.76	42.65
214	5	60.83	65.85	60.41	44.64	1.00	73.26	85.54	9.76	42.39
217	1	46.86	71.29	52.37	59.45	15.58	72.33	72.77	41.46	45.79
217	2	53.63	71.51	56.69	69.78	16.58	74.25	73.08	48.78	49.91
217	3	46.86	71.29	53.33	59.45	15.58	72.33	72.77	41.46	45.79
217	4	53.63	71.51	57.39	69.78	16.58	74.25	73.08	48.78	49.91
217	5	48.14	67.23	49.87	54.58	12.56	70.08	66.09	41.46	41.64
219	1	60.63	63.61	49.11	44.44	2.51	79.83	75.55	29.27	51.92

Continued on next page

Additional file 3 – continued from previous page

Team	Run	Abbrev.	Family	Formula	Ident.	Multip.	System.	Trivial	Undef.	Novel
219	2	88.99	89.56	78.62	84.80	20.60	94.72	92.02	73.17	82.05
219	3	61.64	73.11	51.38	57.70	18.09	82.70	82.45	41.46	59.19
219	4	21.43	9.83	21.11	11.11	0.00	47.83	51.13	0.00	20.70
219	5	63.83	73.83	52.80	62.77	19.09	84.20	85.36	56.10	62.80
222	1	61.71	73.03	43.42	62.38	6.53	80.25	84.77	46.34	39.16
222	2	72.41	73.83	44.55	69.59	6.53	82.23	88.92	48.78	50.75
222	3	61.89	73.03	43.42	62.38	6.53	80.67	84.85	46.34	39.65
222	4	72.73	73.83	44.52	69.59	6.53	82.58	89.04	48.78	51.29
222	5	61.66	72.56	43.33	62.38	6.53	79.67	84.11	41.46	39.06
225	1	50.78	15.85	32.41	29.63	3.02	64.72	68.90	14.63	42.71
225	2	49.94	19.71	32.59	47.76	3.02	65.57	73.18	56.10	48.33
225	3	54.03	47.71	37.64	62.38	7.04	65.62	74.14	56.10	55.07
225	4	48.29	19.41	31.60	46.78	4.02	64.24	71.59	56.10	48.32
225	5	52.48	47.54	36.57	61.40	7.54	64.31	72.92	56.10	54.79
231	1	64.60	81.69	76.36	51.66	41.71	89.43	86.73	51.22	61.09
231	2	70.95	86.55	82.52	66.28	53.27	92.55	90.09	63.41	68.09
231	3	70.95	86.55	82.49	66.28	53.27	92.45	90.09	63.41	68.01
231	4	62.28	70.87	77.52	61.60	39.70	84.08	76.34	48.78	44.40
231	5	71.25	87.63	83.82	67.06	52.76	93.10	90.32	63.41	68.48
233	1	68.39	79.13	75.98	70.17	45.73	88.09	87.11	43.90	65.59
233	2	63.24	74.54	72.87	55.55	33.67	84.64	83.97	39.02	59.97
233	3	68.37	79.21	76.85	69.59	51.26	88.44	87.32	48.78	64.59
233	4	63.61	74.90	73.08	58.48	31.66	84.87	84.44	31.71	59.55
233	5	68.39	78.82	76.59	71.54	49.25	88.39	87.30	48.78	64.70
238	1	66.59	68.58	41.77	69.59	8.04	65.13	79.28	58.54	49.98
238	2	72.88	75.12	45.05	75.63	23.11	67.60	81.28	73.17	57.15
238	3	73.96	74.52	46.30	74.85	20.10	67.19	81.43	70.73	56.23
238	4	62.67	71.15	42.52	65.89	24.62	66.71	78.37	63.41	46.73
238	5	75.73	73.52	46.47	73.49	18.09	65.39	79.71	68.29	57.15
245	1	53.14	64.88	72.12	34.89	43.72	78.45	76.81	31.71	47.81
245	2	55.38	67.53	69.21	36.84	33.67	80.55	77.32	31.71	49.92
245	3	56.54	68.94	72.29	39.38	46.73	80.52	78.62	29.27	52.25
259	1	53.90	59.50	72.76	68.61	27.64	69.57	58.95	51.22	31.68
259	2	55.75	62.20	74.30	70.17	32.16	72.61	62.73	51.22	33.42
259	3	56.74	64.80	74.88	69.98	28.64	74.32	65.48	56.10	33.51
259	4	57.63	66.23	75.46	69.78	31.66	75.61	67.41	58.54	33.94
259	5	58.24	67.28	75.83	69.59	31.66	76.35	68.26	58.54	34.78
262	1	52.33	69.16	58.55	62.38	37.69	80.78	78.55	65.85	57.66
262	2	51.44	74.41	59.72	65.50	33.16	81.13	80.67	36.59	57.77
262	3	52.77	74.49	59.89	65.30	32.66	81.01	80.86	36.59	58.60
262	4	56.79	78.11	61.52	74.46	39.20	82.92	83.03	39.02	61.42
262	5	55.88	78.11	59.08	56.34	37.69	80.96	82.44	51.22	58.04
263	1	63.96	61.54	61.40	38.99	29.14	76.67	75.84	12.20	53.72
263	2	65.48	63.17	61.23	43.08	30.65	79.19	75.88	9.76	56.40
263	3	65.98	65.27	61.72	47.37	35.17	79.92	76.43	17.07	57.98
265	1	36.04	62.53	41.79	53.02	27.14	67.26	65.15	48.78	43.55
265	2	36.19	62.51	42.67	53.02	22.11	67.98	65.37	48.78	43.69
