# Supplementary material for: CHEMDNER: The drugs and chemical names extraction challenge
Source: J Cheminform. 2015 Jan 19;7(Suppl 1):S1. doi: 10.1186/1758-2946-7-S1-S1 (PMC4331685; doi:10.1186/1758-2946-7-S1-S1)
Supplement: Additional file 4 [file 1758-2946-7-S1-S1-S4.pdf]

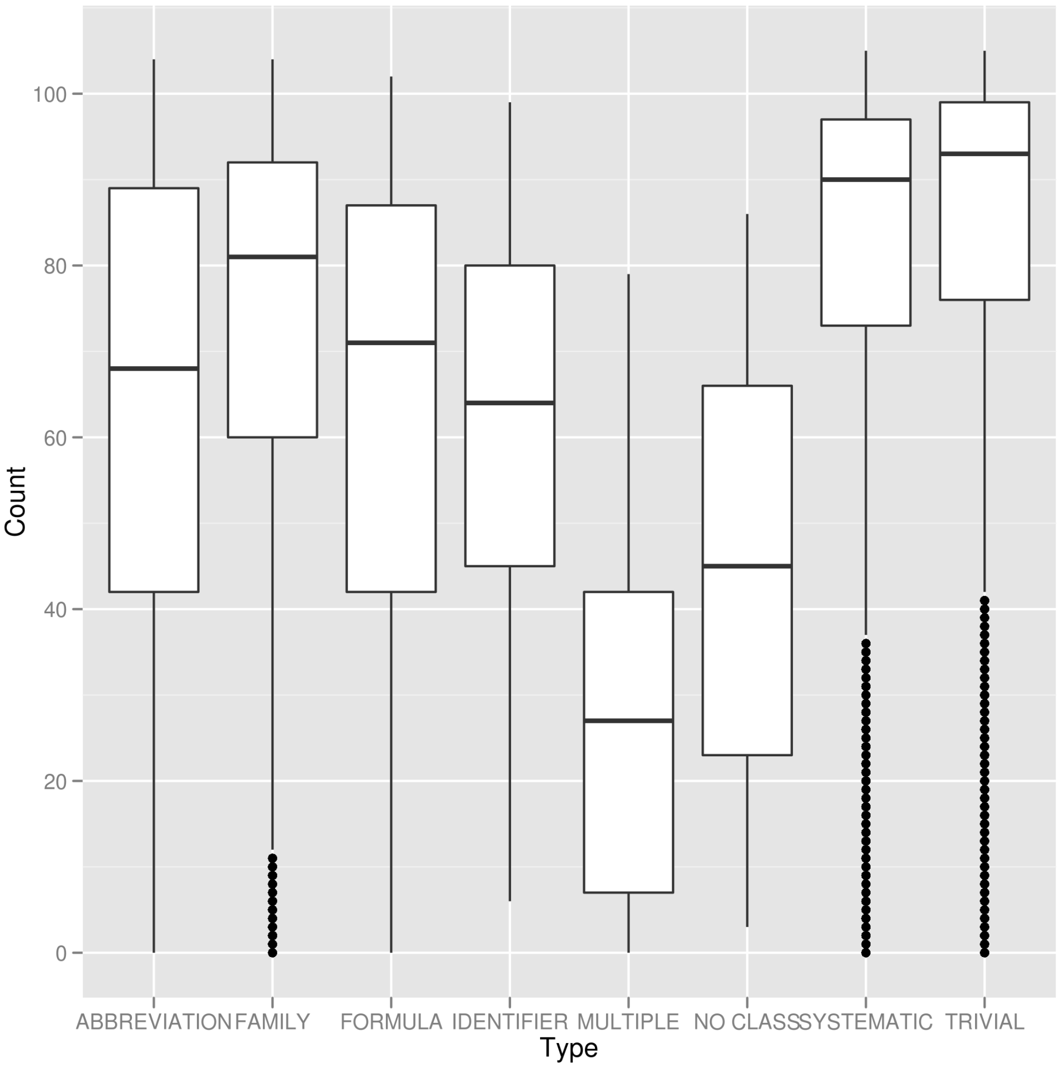

Additional file 4. Boxplot of the number of TP runs for each chemical mention per CEM class. Count: number of runs that correctly identified a mention, Type: CEM class. No class: 41 mentions that could not be assigned unambiguously to one of the CEM classes.
