# Supplementary material for: CHEMDNER: The drugs and chemical names extraction challenge
Source: J Cheminform. 2015 Jan 19;7(Suppl 1):S1. doi: 10.1186/1758-2946-7-S1-S1 (PMC4331685; doi:10.1186/1758-2946-7-S1-S1)
Supplement: Additional file 5 [file 1758-2946-7-S1-S1-S5.pdf]

CEM team rank	1	2	3	4	5	6	7	8	9	10	11	12	13	14	15	16	17	18	19	20	21	22	23	24	25	26
**Lexical resources**																										
ChEBI			*	*	*	*	*				*	*		*				*	*	*						*
PubChem		*	*	*	*						*	*		*							*	*	*			
DrugBank				*			*			*	*	*							*		*					*
UMLS							*		*			*							*						*	*
CTD	*			*		*			*										*							
Jochem				*	*	*						*														
MeSH											*								*							*
ChemSpider												*													*	
Wikipedia			*								*															
Other			*					*	*			*							*				*		*	
**Lexicon expansion**	*		*		*						*	*				*					*				*	*
**Other chem. NER**																										
Oscar4				*	*							*				*	*	*	*	*					*	
ChemSpot	*				*											*	*	*	*		*					*
ChemAxon											*	*		*												
LeadMine			*									*														
MetaMap							*																			*
MiniChem Tagger		*																								
Other											*	*								*		*		*	*	
**BioNLP tools**																										
GeniaTagger				*	*																*					
BANNER	*							*																		
Ab3P	*																									
Other						*		*											*							
**Software**																										
Mallet	*				*	*		*									*	*		*				*		
OpenNLP					*					*	*	*	*					*			*				*	
CRF++	*	*											*		*	*					*	*				
CRFsuite							*			*									*							
Stanford Tokenizer										*													*		*	
Dragon Toolkit	*							*																		
LingPipe				*																		*				
Stanford Parser					*																				*	
Weka										*										*						
SVMLight																			*		*					
NERSuite				*																						
Other							*				*				*	*	*		*				*			*

Additional file 5. Overview of resources used by participating teams.
